# Assessing the Impact of Environment on the Color of Painted Turtles (*Chrysemys picta*) in the Wild

**DOI:** 10.1002/ece3.71702

**Published:** 2025-07-14

**Authors:** Georgina Jaimes, Erik Maki, Beth A. Reinke

**Affiliations:** ^1^ Department of Biology Northeastern Illinois University Chicago Illinois USA

**Keywords:** carotenoid, coloration, crypsis, melanin, painted turtles, phenotypic plasticity, plant density, sexual signaling, water clarity

## Abstract

Animal coloration is a complex phenotype that may be affected by genetics, evolution, ecology, and environment. Disentangling the impact of environment on phenotype can often be done in laboratory studies, but the results do not necessarily correspond to the natural variation present in the wild. Painted turtles are a brightly colored freshwater species that inhabit a variety of environments in North America. There is known to be plasticity in the melanin coloration of the shell of painted turtles in a lab setting, but this has not been measured in the wild. The bright skin coloration that gives painted turtles their name is caused by carotenoids, which can only be obtained from an organism's diet in vertebrates. Though the availability of carotenoids likely varies between environments, and there is evidence that some of the carotenoid‐based coloration in this species is a visual signal, it is unknown if or how environmental variation impacts coloration in the wild. To address this, we measured the effect of the environment on turtle coloration by assessing multiple populations of painted turtles in northern Wisconsin. We measured water clarity and aquatic plant density at each site where turtles were caught. We found that females had brighter carapaces than males, and that plastron brightness varied with water clarity and plant density, despite its ventral orientation. We also found that neither water clarity nor plant density predicted carotenoid chroma, despite reason to believe that light environment and carotenoid availability should impact a visual signal. These findings suggest that colorful phenotypic traits in this turtle species are complex and their potential role as visual signals requires more research. It is crucial to understand the different phenotypes of painted turtles since coloration may influence fitness in this species, and since laboratory studies are unable to represent natural variation.

## Graphical abstract

Painted turtles have bright colors with no known function. We test hypotheses for natural variation in color in this species using visual ecology and signaling theory.

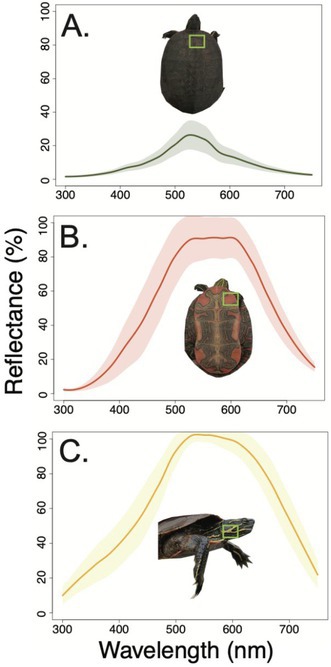

## 1 Introduction

In colorful animal species, it is common for color to vary in brightness or hue within and between populations, but the amount of variation may be dependent on a combination of evolutionary, genetic, physiological, and ecological components (e.g., Siddiqi et al. 2004; Rosenblum 2005; Davis 2014; João et al. 2023; Arbore et al. 2024). Phenotypically plastic coloration is also influenced by environmental factors (Stevens 2016). Disentangling the environmental factors that influence animal coloration in a natural system can be challenging, so this work is often done in a lab where conditions can be controlled and manipulated. However, it is often unknown how these relationships translate to natural variation in wild populations, where environmental variation may not be so discrete.

Adaptive coloration can help animals deter predators, hide from predators, attract mates, out‐compete conspecifics, or communicate with each other, and is thus dependent on the light environment in which it is viewed (Endler 1992). This light environment may in turn be impacted by the scale, season, and vegetation cover. In aquatic systems, the light environment is impacted by vegetation (either indirectly: Scheffer 1999; or directly: e.g., Manatunge et al. 2000) as well as water depth and water clarity/turbidity (Levring and Fish 1956; Seehausen et al. 2008). In clear aquatic conditions, it is especially important that animals can camouflage from potential predators. For example, in the open pelagic zone, many fishes and other aquatic animals have transparent phenotypes or other cryptic mechanisms to better blend with the relatively homogenous environment (McFall‐Ngai 1990). In dark or turbid conditions, visual signals may need to be especially bright or colorful to stand out to receivers (Endler 1992). Additionally, in eutrophic environments, or in water rich in dissolved organic matter, light transmission shifts toward longer wavelengths, like red and orange (Bowling et al. 1986; Kelley et al. 2012). Thus, we may expect to see colors that are used in visual signals similarly shifting to higher wavelengths to enhance transmission. Here again, documenting natural variation across a range of environments is especially important to understand the adaptive consequences and physiological limits of coloration.

Painted turtles, *Chrysemys picta* , are a widespread freshwater turtle species, named for their bright skin and shell coloration. They inhabit a variety of environments in North America, ranging from being eutrophic to oligotrophic, standing water to still water, and roadside ditches to pristine lakes. In the western part of the painted turtle range, the color of the carapace (dorsal shell) can range from olive to black, the plastron (ventral shell) is orange with contrasting black markings, and the neck is covered in bright yellow stripes. The carapace color can primarily be attributed to melanin while the orange and yellow colors are created by carotenoids (Reinke et al. 2017; Steffen et al. 2015).

Painted turtles have been shown to exhibit reversible melanization in a lab environment, plastically lightening or darkening their carapaces to converge with white or black substrates, respectively (Rowe et al. 2009, 2014). However, the extent to which painted turtle carapaces substrate‐match in the wild remains unknown, though the variability of natural water conditions and strong selective pressures at early life stages (Reinke et al. 2017) would suggest that this is a highly relevant and contextual adaptation. For instance, in areas with no vegetation and clear water, substrate matching is likely to be extremely important for this species, while in environments with dense aquatic vegetation or turbid water it may be less so.

In vertebrates, carotenoids can only be obtained from an organism's diet or from maternal reserves (Svensson and Wong 2011). Painted turtles likely obtain the carotenoids that color their plastron and skin stripes from carotenoid‐rich plants and algae, which make up a large part of their diet (Ernst and Lovich 2009). The function of painted turtle skin and shell coloration is unknown, which is especially interesting given the bright ventral coloration of the plastron (Reinke et al. 2017; Steffen et al. 2019). One study has demonstrated that the skin color of *C. picta* is influenced by dietary carotenoid availability in a lab setting (Steffen et al. 2019), but it remains unclear to what extent aquatic vegetation impacts color variation in the wild. Studies in a closely related species ( *Trachemys scripta* ) have identified correlations between immune function and stripe color, suggesting a potential mate selection role for head and/or neck coloration (Ibáñez et al. 2014; Polo‐Cavia et al. 2013). However, in *C. picta* , one study has identified a relationship between the ultraviolet reflectance of neck stripes and innate immune function in one population, but not another (Stasiek and Reinke 2025). Thus, since some of the carotenoid‐based colors may be sexual signals in some populations, their brightness and chroma may be predictably impacted by water clarity. Testing hypotheses about color variation between body patches and in different environments may allow us to disentangle the effects of environment and evolution on color in this species.

Here, we test how water clarity and plant density impact color in natural populations of *C. picta* . We predict that the brightness of the carapace and neck will be negatively correlated with the clarity of the water to enhance crypsis. This relationship may be modulated by plant density since vegetation could provide cover and remove selective pressure. Thus, we expect to find that turtles in clear water are less bright than those in darker water to better blend with substrate, but that thick vegetation (high‐plant density) may remove this effect. Relatedly, we do not expect to find a relationship between plastron brightness and water clarity or plant density because of the ventral orientation of the color. Next, we expect to find that turtles will have increased carotenoid chroma in both the plastron and neck stripes in environments with more aquatic vegetation since carotenoids will be readily available for consumption. However, because neck stripes may be an important sexual signal but there is no evidence that plastrons provide information to conspecifics (there is no known behavioral ventral display in this species, and Stasiek and Reinke 2025 did not find a correlation between immune function and plastron color), we expect that turtles in more turbid water will have higher carotenoid chroma and more red‐shifted spectra in their neck stripes but not their plastrons to enhance signal efficacy in darker environments.

## 2 Methods

### 2.1 Populations

Turtles were captured in June 2020 from six locations within Sawyer County, Wisconsin, using dip‐nets or baited minnow traps. Sites were chosen to sample a diversity of habitats, including a roadside wetland, two eutrophic bays, an oligotrophic lake, a shallow fishing pond, and a flowing channel. Traps had floats which prevented accidental drownings from occurring and were checked two to three times daily. Bait was replaced when needed. To avoid unnecessary recaptures, turtles were temporarily marked on the shells with a small dot of white paint. A maximum of 20 adult turtles (10 females and 10 males) were used per site for the data analysis, but incidentally captured juveniles were also included (Table S1). Each turtle was measured, and its sex was recorded based on secondary sex characteristics, such as gravidity or elongated foreclaws, though juveniles cannot easily be sexed. Every turtle that was captured was released back at the site of capture after data collection was finished. All work with turtles was done with IACUC approval (Protocol number 1119‐1122‐01) and a Wisconsin Department of Natural Resources Scientific Research License (SRLN‐20‐13).

### 2.2 Environmental Variables

Water clarity and aquatic plant density were measured at each body of water where turtles were caught during the capture period in June 2020. Turbidity tubes were used to measure water clarity in nephelometric turbidity units. Water was collected from the approximate middle of where turtles were captured, ensuring that the habitat type was similar to where the turtles were caught (i.e., when caught along a curved shoreline, we sampled in the middle of the shoreline habitat, not in the middle of the lake/pond). Aquatic plant density was measured using a 1 × 1 m quadrat on a 10‐m string. A range of aquatic plant densities within the capture zone was measured by choosing a center point on the shoreline to which the string was anchored and then measuring plant density at nine approximately evenly distributed points from the center point, forming a semicircle (Table S1). The 10‐m distance was chosen because this distance includes most of the emergent vegetation (when present) before deeper water. Turtles were always captured within this vegetation. The number of stems inside each quadrat was counted and then averaged across quadrats per site.

### 2.3 Reflectance

**FIGURE 1:**
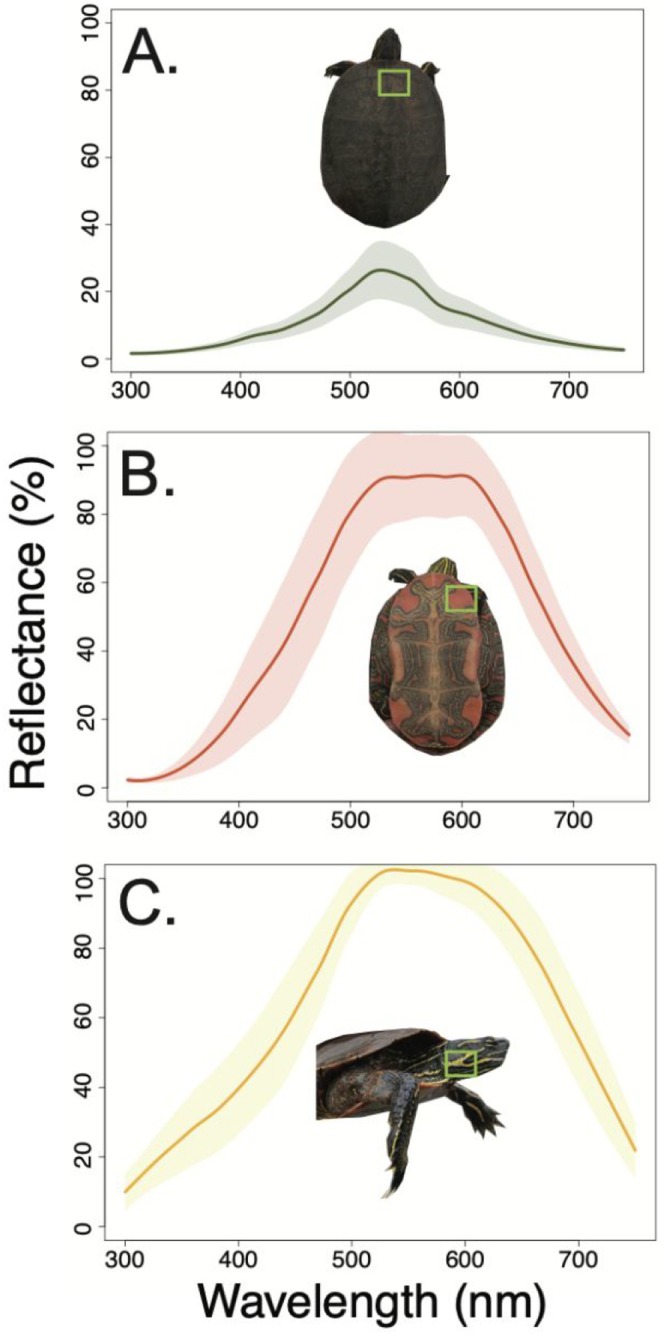
Reflectance spectra of the carapace (A), plastron (B), and neck stripe (C) of the painted turtle. The dark line shows the mean spectrum across all individuals, and the shaded area shows the standard deviation.

An Ocean Insight Flame spectrometer and a PX‐2 Xenon lamp with a bifurcated fiber optic probe were used to measure the reflectance spectra of the brightest spots of a turtle's carapace, plastron, and neck (Figure 1). The integration time was automatically sampled and the boxcar was set to 5. Brightest spots were estimated visually and then verified by moving the probe around until the spectrum was at its brightest (largest area under the curve). The spectrometer was calibrated using a Labsphere reflectance standard that reflects equally (99%) across the ultraviolet and visual spectrum (300–800 nm). The probe was placed at a 45° angle to the surface of the turtle using a probe cover. Three measurements of each body part were taken for each turtle at approximately the same location (using visual inspection of the spectra to ensure that the same color was being measured). Color reflectance data were then visually inspected and analyzed in the R Statistical Programming Language using the “pavo” package (Maia et al. 2013). The *summary* function was used to obtain mean brightness values of the carapace (B2: calculated as the mean relative reflectance across the whole spectrum) and to obtain carotenoid chroma of the plastron and neck stripes (S9: reflectance at 700 nm minus the reflectance at 450 nm divided by 700 nm) for each averaged set of spectra per individual (Maia et al. 2013).

### 2.4 Statistical Analysis

All analyses were performed in the R Statistical Programming Language (R version 3.6.1) using the “lme4” package (Bates et al. 2015). We constructed five linear mixed effects models based on the hypotheses for carapace brightness, plastron brightness, neck stripe brightness, plastron carotenoid chroma, and the neck stripe carotenoid chroma. Water clarity and plant density (and their interaction for the brightness models) were used as fixed effects, with carapace length and sex (male, female, juvenile) as additional fixed effects and population as a random intercept. We did not include the interaction between the two environmental variables for the carotenoid chroma models because we did not have a hypothesis for how they may interact to affect chroma, and they added high levels of multicollinearity to the models. Clarity was log‐transformed to better match the scale of the other predictor variables. Significance testing was done with likelihood ratio tests. Assumptions of linear mixed effects models were checked before completing the analyses using the 'performance' package (Lüdecke et al. 2020; Appendix S1).

## 3 Results

The reflectance spectra of the carapace, plastron, and neck varied in shape, with the plastron showing the most variation across individuals and across wavelengths (Figure 1). Neck stripes especially varied between 350 and 450 nm, and the carapace spectrum varied in the height (intensity) of the peak (Figure 1).

### 3.1 Effects of Environment on Brightness

**FIGURE 2:**
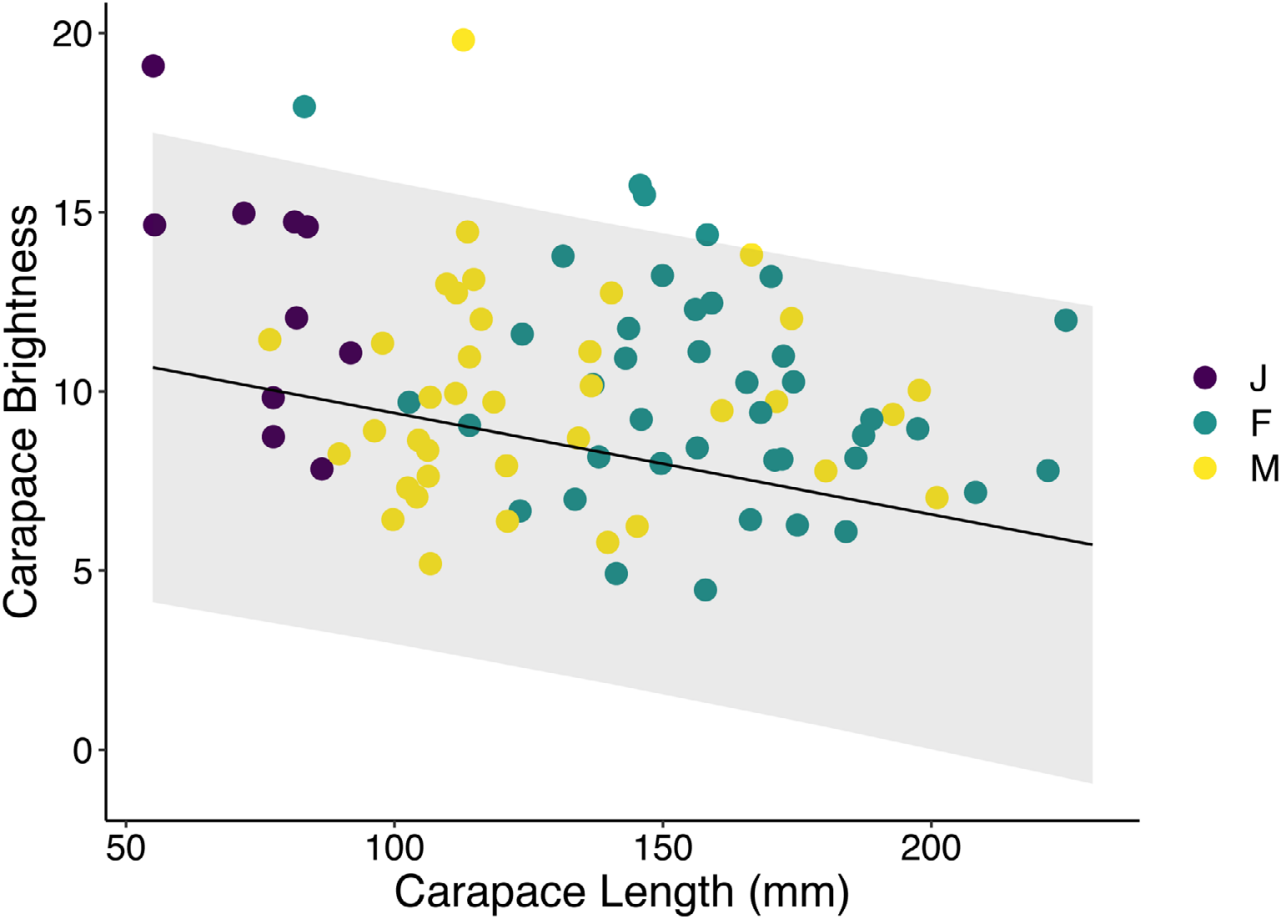
Carapace brightness decreases as carapace length increases. Juveniles are denoted with purple points, females with yellow points, and males with green points. The regression line shows the estimate from the linear mixed effects model with population as a random effect and log‐transformed water clarity, the interaction between log‐transformed clarity and plant density, carapace length, and sex as fixed effects.

Both sex and carapace length significantly predicted carapace brightness, with larger turtles (i.e., older turtles) having lower carapace brightness than smaller (younger) turtles (*χ* ^2^ = 10.49, *p* = 0.001; Table 1, Figure 2). Female carapace brightness significantly differed from male and juvenile brightness (*χ* ^2^ = 6.61, *p* = 0.04; Table 1). Females had darker carapaces than males, while juveniles had the lightest carapaces (Figure 2). To disentangle the effects of carapace length and sex (painted turtles are sexually dimorphic with females reaching a much larger size than males), we subset the data by sex and performed linear mixed effects models with just carapace length as a fixed effect and population as a random effect. In both females (*χ* ^2^ = 1.58, *p* = 0.21) and males (*χ* ^2^ = 1.94, *p* = 0.16), length did not affect brightness, suggesting the difference is largely due to sex independent of size. Juvenile carapace length did predict brightness (*χ* ^2^ = 4.77, *p* = 0.03), but since juveniles cannot accurately be sexed, this relationship could still be driven by sex differences.

**TABLE 1:** Model estimates testing the effect of environment, carapace length, and sex (juvenile, male, female) on the brightness of the carapace (top), plastron (middle) and neck stripes (bottom).

Carapace brightness				
Fixed effects	Estimates	SE	*t*	*p*
Intercept	23.293	5.475	4.254	
Log water clarity	−1.209	0.900	−1.343	0.129
Log clarity × plant density	−0.051	0.034	−1.513	0.061
Carapace length	−0.028	0.009	−3.156	**0.001**
Sex: juveniles	0.944	1.061	−1.597	**0.037**
Sex: males	−0.923	0.578	−1.597	
**Random**	**Variance**	**SD**	***N***	
**Intercept**				
Population	4.798	2.190	6	
**Plastron brightness**				
**Fixed effects**	**Estimates**	**SE**	***t***	***p***
Intercept	87.798	11.016	7.970	
Log water clarity	−4.200	1.371	−3.062	**0.005**
Log clarity × plant density	−0.095	0.055	−1.715	**0.048**
Carapace length	−0.083	0.034	−2.413	**0.013**
Sex: juveniles	−3.254	4.133	−0.787	0.640
Sex: males	−1.620	2.234	−0.725	
**Random**	**Variance**	**SD**	***N***	
**Intercept**				
Population	76.413	8.741	6	
**Neck brightness**				
**Fixed effects**	**Estimates**	**SE**	***t***	***p***
Intercept	49.867	7.288	6.842	
Log water clarity	1.536	0.740	2.074	0.086
Log clarity × plant density	0.004	0.032	0.111	0.908
Carapace length	0.060	0.026	2.277	**0.020**
Sex: juveniles	−0.669	3.200	−0.209	0.491
Sex: males	−1.893	1.739	−1.089	
**Random**	**Variance**	**SD**	***N***	
**Intercept**				
Population	46.82	6.842	6	

*Note:* *p*‐values were calculated with a likelihood ratio test; significant *p*‐values (*p* < 0.05) are bolded. Note that *p*‐values for sex (categorical variable) are for the entire variable.

We also found that carapace length influenced both plastron brightness and neck stripe brightness, though in opposite directions. Larger turtles had less bright plastrons (*χ* ^2^ = 6.17, *p* = 0.013; Table 1) but brighter neck stripes (*χ* ^2^ = 5.39, *p* = 0.020; Table 1), independent of sex. Additionally, both water clarity and the interaction between water clarity and plant density influenced plastron brightness (Table 1). Turtles in clearer water had less bright plastrons (*χ* ^2^ = 8.01, *p* = 0.005), and there was a significant interaction between water clarity and vegetation density (*χ* ^2^ = 3.91, *p* = 0.05). To further investigate this interaction, we analyzed the two sites with the clearest water separately since we hypothesized that in clear water with low vegetation, we should see less bright turtles than in clear water with high vegetation, and fitted a model that included carapace length and the random effect of population. We found that in the clearest water, plant density is significantly negatively correlated with brightness (*χ* ^2^ = 4.99, *p* = 0.03; Table 2).

**TABLE 2:** Model estimate testing the effect of plant density and carapace length on the plastron carotenoid chroma for only the two sites with the highest water clarity (MB and SB).

Fixed effects	Estimates	SE	*t*	*p*
Intercept	71.586	8.017	8.929	
Plant density	−1.737	0.575	−3.019	**0.025**
Carapace length	−0.041	0.037	−1.098	0.275
**Random**	**Variance**	**SD**	***N***	
**Intercept**				
Population	80.050	8.947	2	

*Note:* *p*‐values were calculated with a likelihood ratio test; significant *p*‐values (*p* < 0.05) are bolded.

### 3.2 Effects of Environment on Carotenoid Chroma

**FIGURE 3:**
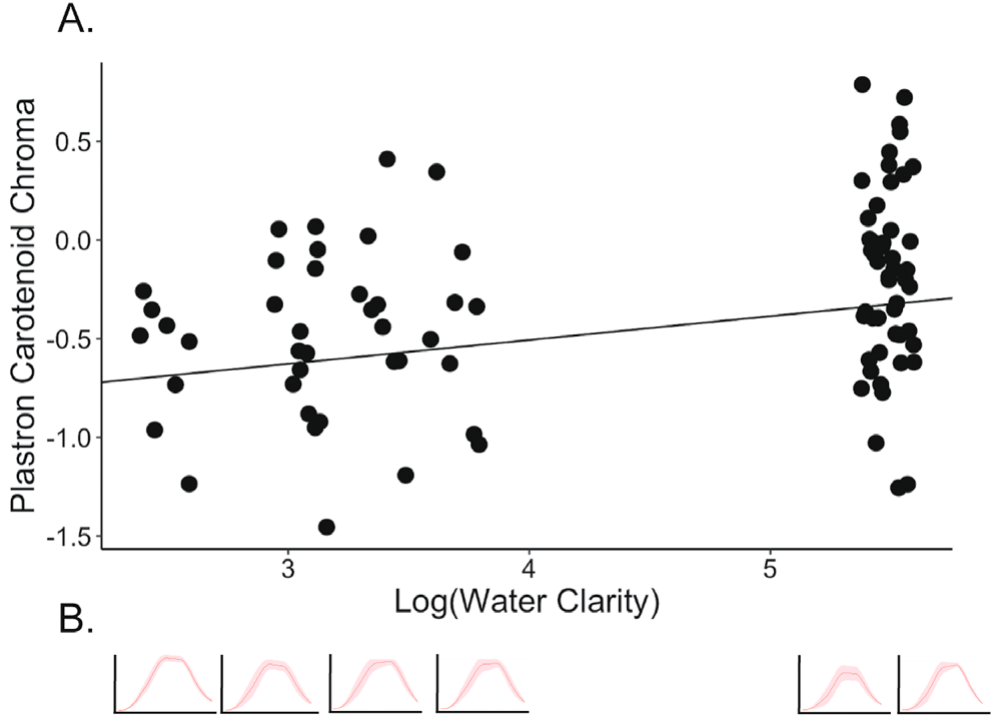
Plastron carotenoid chroma did not change with water clarity or any other fixed effects (A), though the shape of the spectra shifted slightly with water clarity (B). The regression line shows the estimate from the linear mixed effects model with population as a random effect and log‐transformed water clarity, plant density, their interaction, carapace length, and sex as fixed effects. Points are jittered along the *x*‐axis for clarity. The mean reflectance spectrum is shown for each site, with the standard deviation shaded. The *x*‐axis is from 300 to 750 nm and the *y*‐axis is from 0% to 100% reflectance for all spectra.

None of our fixed effects (water clarity, plant density, carapace length, or sex) influenced the carotenoid chroma of the plastron or the neck stripes (Table 3; Figure 3a). However, we saw some change in the shape of the plastron spectra with increased water clarity (Figure 3b). To test if this is a real pattern, we ran an additional two linear fixed effects models to test if water clarity, with population as a random effect, affects the wavelength of maximum reflectance on the plastron and the neck stripes. We found no effect of water clarity (*χ* ^2^ = 0.22, *p* = 0.64) on the neck stripe wavelength, but a slight positive effect of water clarity on the wavelength of maximum reflectance on the plastron (*χ* ^2^ = 3.65, *t* = 1.84, *p* = 0.06).

**TABLE 3:** Model estimates testing the effect of environment, carapace length, and sex (juvenile, male, female) on the plastron carotenoid chroma (top) and neck stripe carotenoid chroma (bottom).

Plastron carotenoid chroma				
Fixed effects	Estimates	SE	*t*	*p*
Intercept	−0.990	0.557	−1.777	
Log water clarity	0.121	0.069	1.751	0.072
Plant density	−0.001	0.007	−0.212	0.826
Carapace length	0.001	0.002	0.422	0.662
Sex: juveniles	0.043	0.210	0.204	0.575
Sex: males	0.111	0.114	0.968	
**Random**	**Variance**	**SD**	***N***	
**Intercept**				
Population	0.204	0.451	6	
**Neck carotenoid chroma**				
**Fixed effects**	**Estimates**	**SE**	***t***	***p***
Intercept	0.396	0.565	0.700	
Log water clarity	−0.081	0.074	−1.097	0.193
Plant density	−0.003	0.007	−0.392	0.629
Carapace length	−0.001	0.002	−0.442	0.653
Sex: juveniles	−0.266	0.203	−1.311	0.191
Sex: males	−0.183	0.002	−0.442	
**Random**	**Variance**	**SD**	***N***	
**Intercept**				
Population	0.189	0.434	6	

*Note:* *p*‐values were calculated with a likelihood ratio test; significant *p*‐values (*p* < 0.05) are bolded. Note that *p*‐values for sex (categorical variable) are for the entire variable.

## 4 Discussion

We tested five predictions about the relationship between brightness or carotenoid chroma and environmental variables. These predictions were based on previous lab work with this species, known patterns of sexual selection in the species, and visual ecology theory. In almost all cases, our results did not match our predictions, and in some cases, were completely contrary to what was expected.

Contrary to our predictions that carapace brightness would be negatively correlated with water clarity with a possible interactive effect between clarity and plant density, carapace brightness was driven primarily by sex, and not by water clarity or plant density. Previous work has shown that painted turtles in the lab exhibit reversible melanization to match substrate coloration (Rowe et al. 2009), but it is possible that there is not enough variation in the natural visual environment to detect differences between populations. In fact, a study that compared the presence of reticulate melanism in painted turtles across three different habitats (lake, river, and wetlands) found no correlation with habitat type (though they weren't looking specifically at substrate color; Gronke et al. 2006). However, in spiny softshell turtles in the wild, the red and green color values of the turtle's carapace were positively correlated with the habitat's bottom substrate values (McGaugh 2008).

Changes in carapace brightness with age have been documented in other species of freshwater turtles, including big‐headed turtles ( *Platysternon megacephalum* ) (Cao et al. 2019), red‐eared sliders ( *Trachemys scripta* ) (Cao et al. 2018), three‐toed box turtles ( *Terrapene carolina triunguis* ) (Leuck and Carpenter 1981) and European pond turtles ( *Emys orbicularis* ) (Ibáñez et al. 2017) though the mechanisms or functions of these changes are not known in most cases. We identified the same pattern in *C. picta* (larger turtles had darker carapaces than smaller ones) but determined that this pattern is largely driven by sex differences, where females are darker than males. Sexual dichromatism in carapace brightness has not been identified in this species previously, despite other studies testing specifically for sex differences in carapace brightness (Stasiek and Reinke 2025; Steffen et al. 2021). Stasiek and Reinke (2025) more intensively sampled one of the populations included in this paper (Musky Bay) using the same reflectance protocols, but detected no differences in carapace brightness. Thus, sexual dichromatism in brightness may vary by population, a conclusion supported by the interpopulational differences identified in that same study (Stasiek and Reinke 2025).

Similar to the carapace prediction, we predicted that neck stripe brightness should decrease with water clarity and potentially be modulated by plant density, in order to maximize crypsis. Relatedly, we predicted that plastron brightness should not be affected by either water clarity or plant density because of its ventral orientation. However, we found results that did not match these predictions at all. Neck stripe brightness was not impacted by either of the environmental variables, but did increase with size independent of sex (Table 1). Plastron brightness decreased with size, but additionally was negatively correlated with both water clarity and the interaction between water clarity and plant density. When we further explored the interactive effect to isolate the conditions we predicted for cryptic colors (where turtles in clear water with low vegetation should be darker than turtles in clear water with high vegetation), we found the opposite pattern. Turtles in clear water and low plant density actually had brighter plastrons than those in clear water and high plant densities (Table 2). This may be partially explained if the presence of high plant densities results in increased carotenoids in the tissue. There is indeed a strong negative correlation between brightness and carotenoid chroma of the plastron; however, we did not find that plant density impacted carotenoid chroma at all (Table 3).

We did not find that neck stripe brightness was affected by environment, despite another study identifying evidence that the neck stripe may be an honest signal (Stasiek and Reinke 2025), thus indicating that we should expect to see neck stripes having predictable relationships to environmental light variables (Endler 1992). We predicted that the carotenoid chroma, the colorimetric that has been correlated with innate immune function in this species, would decrease with water clarity to enhance signal efficacy. However, we found that the carotenoid chroma of the neck stripe was not correlated with either of the environmental variables, nor with the size of the turtle, nor with the sex. Stasiek and Reinke (2025) more intensively sampled one (Musky Bay) of the six populations used in this study, and found no correlation with immunity, suggesting that the neck stripes are not honest signals in Musky Bay. However, Stasiek and Reinke (2025) only sampled two populations, and since they found evidence for honest signaling in one but not the other, we felt that our hypothesis about neck stripe brightness being correlated with environment to enhance signal efficacy was still worth investigating. The lack of a correlation between neck stripe brightness and environment suggests that the stripes may not be signals in more than one of the populations included here—or at least in enough to dilute any detectable trend. However, in the population where signal honesty was detected by Stasiek and Reinke (2025), the painted turtles sometimes have red neck stripes among their yellow stripes. This is not the case with any of the six populations included in this study, and so differences in honest signaling may be driven by broader geographical patterns.

The carotenoid chroma of the plastron also did not change with plant density, contrary to our prediction. The plastron is colored by carotenoids (Reinke et al. 2017) a pigment abundant in plants and algae. We predicted that a higher availability of carotenoids would result in a higher carotenoid chroma in the plastron. Painted turtles are opportunistic omnivores, meaning plants only make up a component of their diet (Ernst and Lovich 2009) and the degree of herbivory appears to vary across the species range (Cooley et al. 2003; Padgett et al. 2010). In the same species, Steffen et al. (Cooley et al. 2003; Padgett et al. 2010) found that turtles supplemented with lutein (a yellow carotenoid) in addition to a commercial pellet or gel formula in the lab exhibited increased red chroma in the red areas on the underside of the carapace. It is not clear how the levels of carotenoid chosen for that lab experiment compare to the levels naturally consumed by painted turtles in the wild since that has never been measured, and so it is possible that they contained significantly higher amounts of carotenoid that would be consumed even in an environment with high‐plant density. Additionally, though the plastrons were not measured in that study (their population has yellow plastrons, rather than the bright orange plastrons of the populations in this study), the mechanisms controlling the red on the underside of the carapace are likely the same or similar to those controlling the red/orange of the plastron, so it is reasonable to expect that increased availability of carotenoids in the wild should lead to increased carotenoid chroma. However, it is possible that because the red components of the plastron (and underside of the carapace) make up much more surface area of the shell in these populations than in those studied by Steffen et al. (2019), any change in red chroma is much more diffuse in our populations.

The exact composition of types of carotenoids available in the wild, and consumed by painted turtles, is not known. The identification of specific types of carotenoids from reflectance or absorbance spectra is complicated by the fact that multiple types are often used in conjunction with each other, and they may have differing functions or sources (Andersson et al. 2007). The variation in reflectance shape and peaks of plastrons that we observed across sites (Figure 3b), while not identifiable to type of carotenoid, suggests that (1) there is variance in selective pressure on the plastron caused by the light environment of the water body, (2) different amounts or types of carotenoids may be present at different sites, or (3) both of these factors contribute to the observed variance. Because plant density and water clarity were correlated at our two sites (the clearest sites had the lowest plant density), it is especially hard to disentangle these factors. Other studies have found that in highly eutrophic or turbid environments, the light spectrum is red‐shifted (Bowling et al. 1986; Kelley et al. 2012). We found that as water clarity increased, the wavelength of maximum reflectance on the plastrons became slightly more red. This is the opposite pattern of what would be predicted for a visual signal, providing additional evidence that the plastron coloration likely does not have a signaling function (Reinke et al. 2017). The dominant vegetative species at the high‐plant density sites were *Nuphar lutea* (yellow water‐lily) and *Elodea canadensis* (American waterweed). The dominant vegetation in low‐density plant sites was typically true sedges (tall grass) and *Potamogeton natans* (floating leaf pondweed). The differences in dominant vegetation types may result in differences in the types of available carotenoids in the environment. However, plastron coloration has previously been found to vary widely even within a single population and environment (Surasinghe et al. 2019) and so it is extremely difficult to test this hypothesis without biochemical methods.

It is essential to understand how lab manipulations of coloration translate to natural variation in wild populations. Despite clear patterns of carapace brightness plasticity having been previously identified in this species in a lab environment (Rowe et al. 2009, 2014), we found no variation in brightness between populations that could be attributable to water clarity or vegetation density. Despite carotenoid supplementation in a lab environment having a clear impact on neck stripes and parts of the shell, we found variation in carotenoid chroma that could not easily be attributed to the environment. We did not find support for our predictions based on visual ecology, such that the carotenoid chroma of the neck stripes, thought to be an honest signal in at least some populations, does not increase in less clear waters. This again may be attributable to under‐documented natural variation in signal honesty and color variation. This study demonstrates that disentangling the effects of the environment on coloration, especially phenotypically plastic coloration, can be extremely difficult but is necessary to understand the true extent of natural variation in animal coloration.

## Author Contributions

**Georgina Jaimes:** conceptualization (supporting), data curation (equal), formal analysis (equal), investigation (equal), methodology (equal), visualization (equal), writing – original draft (lead), writing – review and editing (equal). **Erik Maki:** investigation (equal), methodology (equal), writing – review and editing (equal). **Beth A. Reinke:** conceptualization (lead), data curation (equal), formal analysis (equal), funding acquisition (lead), investigation (equal), methodology (equal), project administration (lead), resources (equal), supervision (lead), visualization (equal), writing – original draft (equal), writing – review and editing (equal).

## Conflicts of Interest

The authors declare no conflicts of interest.

## Supporting information


Appendix S1

## Data Availability

All the required data are uploaded as Supporting Information.
