# Supplementary material for: Assessing the Impact of Environment on the Color of Painted Turtles (Chrysemys picta) in the Wild
Source: Ecol Evol. 2025 Jul 14;15(7):e71702. doi: 10.1002/ece3.71702 (PMC12256770; doi:10.1002/ece3.71702)

**Appendix 1**: Model Assumption Graphs

All graphs created with the *check_model* function of the ‘*performance*’ package (Lüdecke et al., 2020).

Hypothesis 1: Carapace Brightness ~ Log(Water Clarity) +Log(Water Clarity) : Plant Density + Length + Sex + (1|Population)


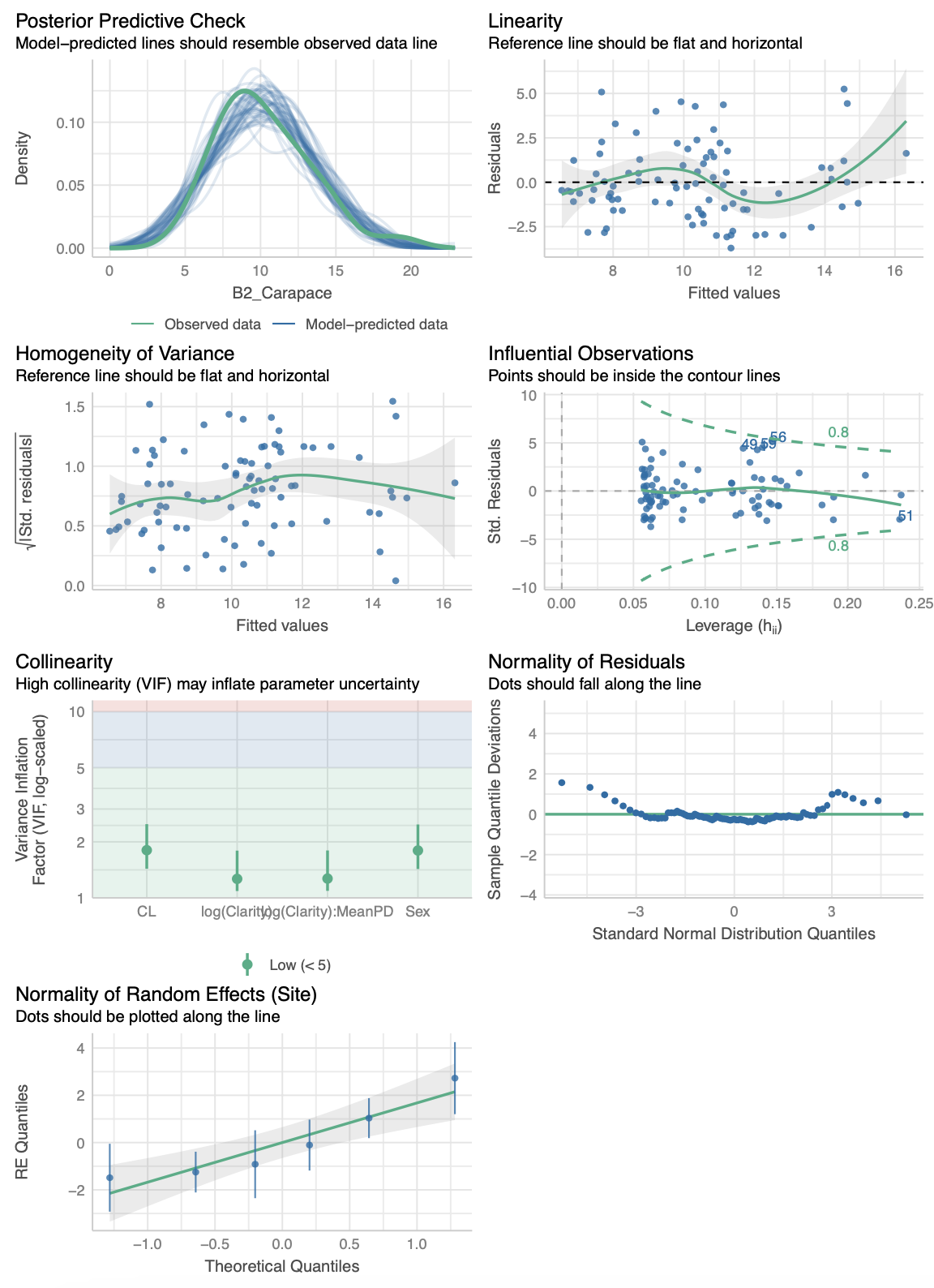


Hypothesis 2: Plastron Brightness ~ Log(Water Clarity) + Log(Water Clarity) : Plant Density + Length + Sex + (1|Population)

Hypothesis 3: Neck Stripe Brightness ~ Log(Water Clarity) + Log(Water Clarity) : Plant Density + Length + Sex + (1|Population)

Hypothesis 4: Plastron Carotenoid Chroma ~ Log(Water Clarity) + Plant Density + Length + Sex + (1|Population)

Hypothesis 5: Neck Stripe Carotenoid Chroma ~ Log(Water Clarity) + Plant Density + Length + Sex + (1|Population)

Table S1. Summary statistics for sex distribution of *Chrysemys picta* from each location and environmental variables for each location sampled in Sawyer County, WI.

|  | Number of Juveniles | Number of Females | Number of Males | Clarity | Plant Density Mean | Plant Density St. Dev. |
| --- | --- | --- | --- | --- | --- | --- |
| Stuckey Bay | 3 | 10 | 11 | 240 | 13.22 | 4.18 |
| Musky Bay | 5 | 10 | 10 | 240 | 8.78 | 5.63 |
| River Bridge | 0 | 3 | 7 | 40 | 4.67 | 7.48 |
| Little Grindstone | 0 | 10 | 5 | 21 | 35.56 | 21.09 |
| Little Stone | 0 | 5 | 4 | 30 | 18.78 | 10.26 |
| Wetland Road | 3 | 3 | 4 | 12 | 42.22 | 14.26 |


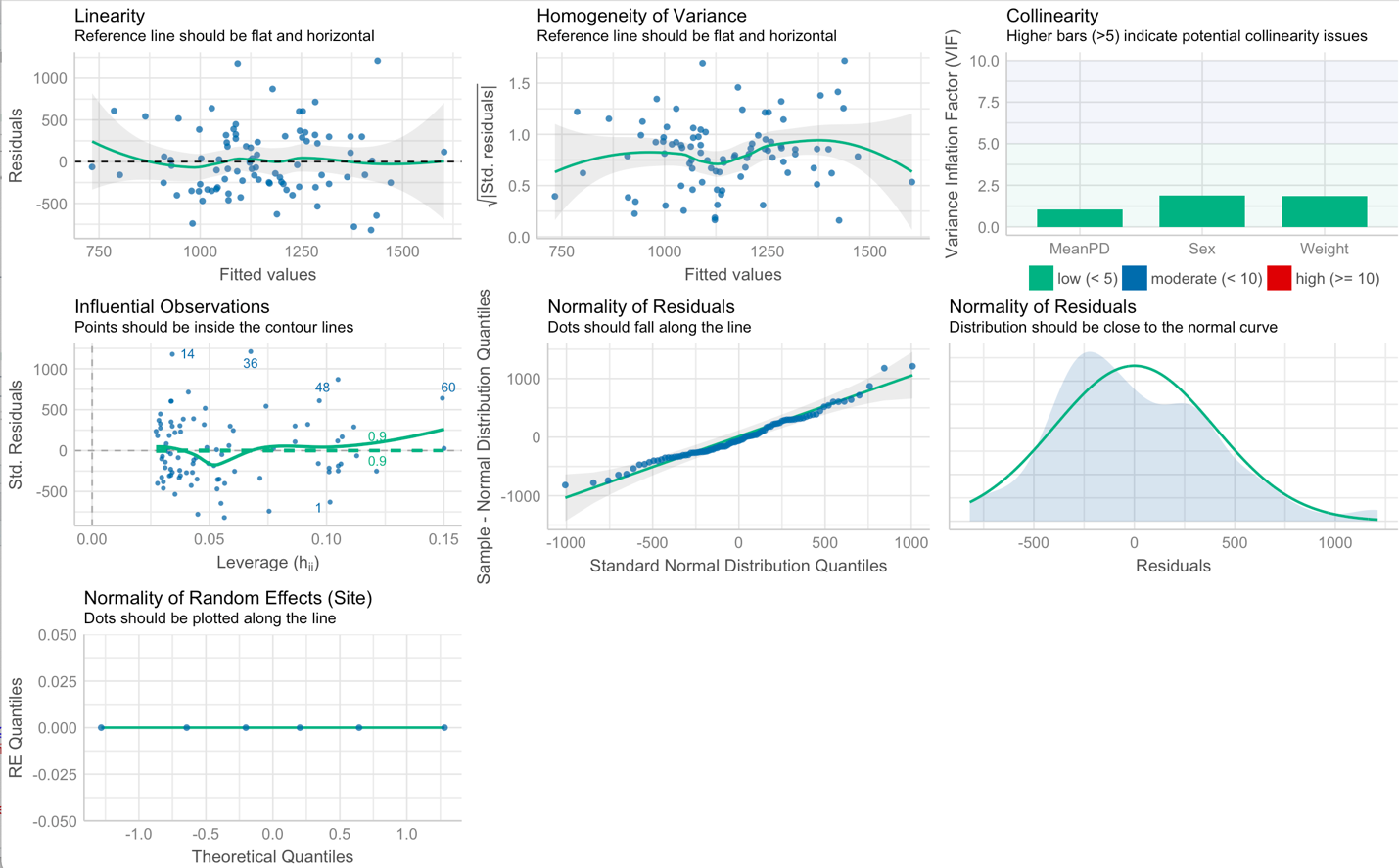

Supplement: Supplementary file 1 — Appendix S1 [file ECE3-15-e71702-s001.docx]
